# Prognostic analysis of high-flow nasal cannula therapy and non-invasive ventilation in mild to moderate hypoxemia patients and construction of a machine learning model for 48-h intubation prediction—a retrospective analysis of the MIMIC database

**DOI:** 10.3389/fmed.2024.1213169

**Published:** 2024-03-01

**Authors:** Wei Fu, Xiaoqing Liu, Lili Guan, Zhimin Lin, Zhenfeng He, Jianyi Niu, Qiaoyun Huang, Qi Liu, Rongchang Chen

**Affiliations:** ^1^State Key Laboratory of Respiratory Disease, National Clinical Research Center for Respiratory Disease, Guangzhou Institute of Respiratory Health, the First Affiliated Hospital of Guangzhou Medical University, Guangzhou, Guangdong, China; ^2^Department of Critical Care Medicine, the First Affiliated Hospital of Guangzhou Medical University, Guangzhou Institute of Respiratory Health, Guangzhou, China; ^3^Emergency Intensive Care Department, the First Affiliated Hospital of Zhengzhou University, Zhengzhou, Hena, China

**Keywords:** high flow nasal cannula, non-invasive ventilation, ICU 28-day mortality, 48-h intubation risk predictive model, maching learning

## Abstract

**Background:**

This study aims to investigate the clinical outcome between high-flow nasal cannula (HFNC) and non-invasive ventilation (NIV) therapy in mild to moderate hypoxemic patients on the first ICU day and to develop a predictive model of 48-h intubation.

**Methods:**

The study included adult patients from the MIMIC III and IV databases who first initiated HFNC or NIV therapy due to mild to moderate hypoxemia (100 < PaO2/FiO2 ≤ 300). The 48-h and 30-day intubation rates were compared using cross-sectional and survival analysis. Nine machine learning and six ensemble algorithms were deployed to construct the 48-h intubation predictive models, of which the optimal model was determined by its prediction accuracy. The top 10 risk and protective factors were identified using the Shapley interpretation algorithm.

**Result:**

A total of 123,042 patients were screened, of which, 673 were from the MIMIC IV database for ventilation therapy comparison (HFNC *n* = 363, NIV *n* = 310) and 48-h intubation predictive model construction (training dataset *n* = 471, internal validation set *n* = 202) and 408 were from the MIMIC III database for external validation. The NIV group had a lower intubation rate (23.1% vs. 16.1%, *p* = 0.001), ICU 28-day mortality (18.5% vs. 11.6%, *p* = 0.014), and in-hospital mortality (19.6% vs. 11.9%, *p* = 0.007) compared to the HFNC group. Survival analysis showed that the total and 48-h intubation rates were not significantly different. The ensemble AdaBoost decision tree model (internal and external validation set AUROC 0.878, 0.726) had the best predictive accuracy performance. The model Shapley algorithm showed Sequential Organ Failure Assessment (SOFA), acute physiology scores (APSIII), the minimum and maximum lactate value as risk factors for early failure and age, the maximum PaCO_2_ and PH value, Glasgow Coma Scale (GCS), the minimum PaO_2_/FiO_2_ ratio, and PaO_2_ value as protective factors.

**Conclusion:**

NIV was associated with lower intubation rate and ICU 28-day and in-hospital mortality. Further survival analysis reinforced that the effect of NIV on the intubation rate might partly be attributed to the other impact factors. The ensemble AdaBoost decision tree model may assist clinicians in making clinical decisions, and early organ function support to improve patients’ SOFA, APSIII, GCS, PaCO_2_, PaO_2_, PH, PaO_2_/FiO_2_ ratio, and lactate values can reduce the early failure rate and improve patient prognosis.

## Introduction

Acute hypoxemia is a common phenomenon in intensive care unit (ICU) daily clinical practice and is caused by a wide range of etiologies, including acute respiratory distress syndrome, pulmonary infection, sepsis, multiple organ dysfunction syndrome, and exacerbation of chronic pulmonary and heart disease. In the SPECTRUM study, the incidence of hypoxemia was 54% among all ICU patients with all types of oxygenation devices (1). The presence of hypoxemia has been widely demonstrated to be associated with higher mortality (2–5), ICU length of stay (6), and longer mechanical ventilation duration (7).

High-flow nasal cannula (HFNC) and non-invasive ventilation (NIV) are two widely accepted non-invasive methods of respiratory support used in ICU daily clinical practice for improvement in gas exchange and ventilation and even play an important role in resource-constrained COVID-19 (8, 9). Recent guidelines have recommended HFNC as the optimal first-line therapy for acute hypoxemia respiratory failure based on the physiological and clinical effects and better patient compliance (10). However, the evidence for this suggestion is inconsistent and imprecise due to different experimental conditions and evaluation criteria in existing studies (10). Therefore, the superior non-invasive respiratory support therapy is still under debate. It remains difficult and confusing for clinicians, especially in the emergency room and ICUs, to determine optimal strategies for acute hypoxemia without a clear cause.

The prominent advantage of both oxygen therapies is their effect on avoiding invasive ventilation-related complications associated with unnecessary endotracheal intubation and sedation. However, recent research has demonstrated that excess spontaneous inspiratory effort could result in high transpulmonary pressure fluctuation (11) and large total lung strain (12, 13) and finally lead to additional lung injury associated with treatment failure (14), especially when NIV therapy is coupled with high tidal volume (15) and rapid respiratory rate (16). Therefore, identifying predictive risk factors and modeling treatment failure may facilitate the early identification of high-risk patients and improve clinical decision-making and outcomes.

To investigate whether NIV therapy in mild to moderate hypoxemia of the whole clinical spectrum is associated with lower mortality and intubation rate compared with HFNC, we performed a retrospective research study based on the Medical Information Mart for Intensive Care III and IV (MIMIC-III, IV) (17, 18). We also performed survival analysis to compare the 48-h and 30-day intubation rates between two groups and constructed a 48-h intubation risk model to assist professional clinicians in making clinical decisions on ventilation therapy options for acute hypoxemic patients.

## Materials and methods

### General information and ethics

This retrospective study was conducted based on the MIMIC database, a large and single-center database comprising information relating to patients admitted to critical care units at Beth Israel Deaconess Medical Center (BIDMC), Boston, Massachusetts United States. One author (WF) finished the training course and signed the data use agreement to obtain access to the database for data extraction. The use of the MIMIC-III database was approved by the Institutional Review Boards of BIDMC and MIT, and a waiver of informed consent was granted.

### Study population

**Figure 1:**
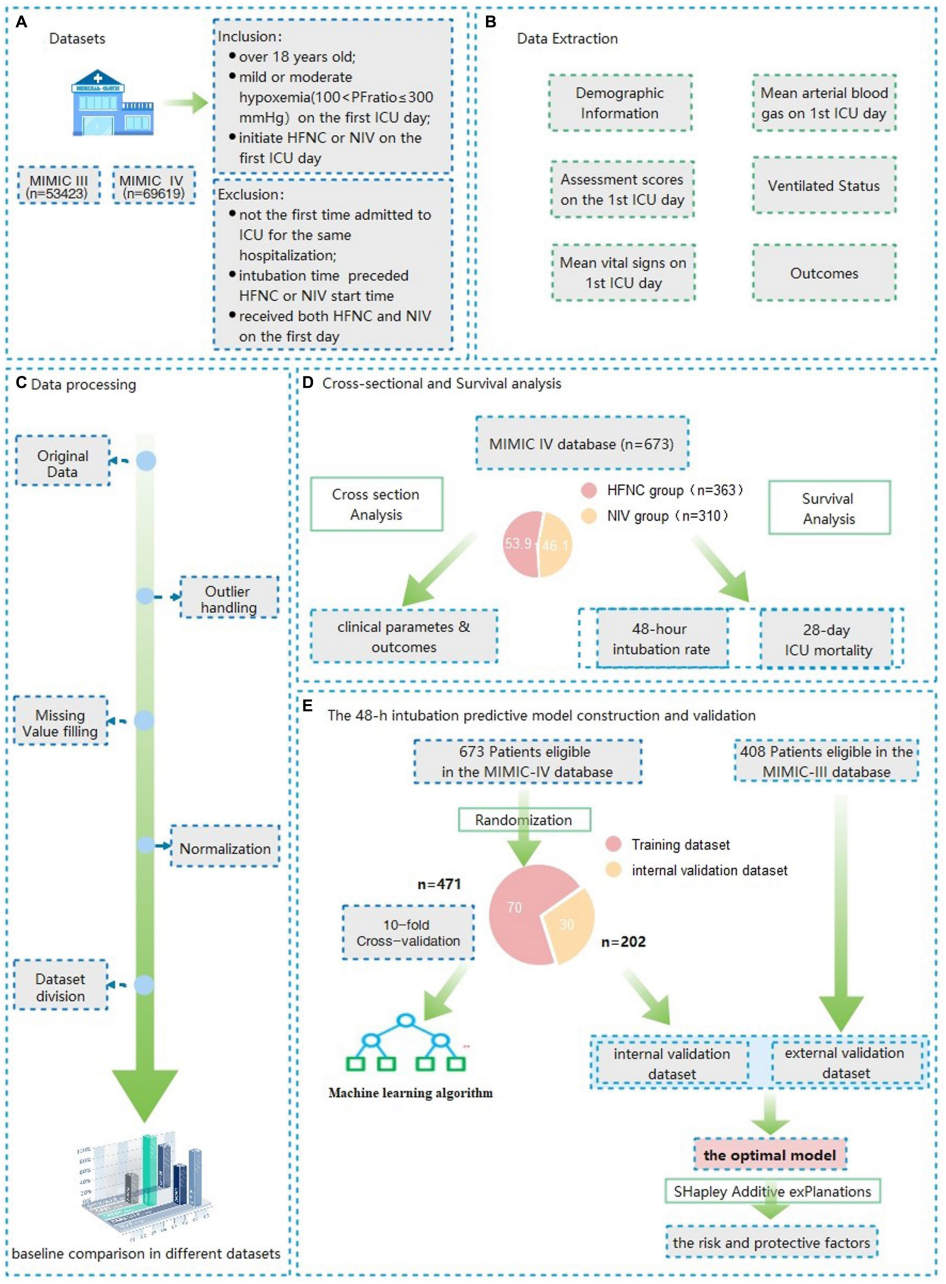
Flow charts of the data extraction, model construction and validation of Patients with mild to moderate hypoxemia at the first ICU admission day.

All patients admitted to an ICU from 2008 to 2019 in the MIMIC IV database were screened to explore the prognostic analysis between HFNC and NIV therapy and 48-h intubation predictive model construction. The eligible patients extracted from the MIMIC III database from 2001 to 2008 were established as the validation cohort for the predictive model external validation (Figure 1). The detailed inclusion criteria were as follows: over 18 years old; with mild or moderate hypoxemia (100 < PaO_2_/FiO_2_ ≤ 300) during the first ICU day; initiated HFNC or NIV on the first ICU day. The exclusion criteria were as follows: not the first time admitted to the ICU for the same hospitalization; intubation time preceded HFNC or NIV start time; received both HFNC and NIV on the first day.

### Data extraction

The following data of the study subjects were extracted from the MIMIC database: gender, age, body mass index (BMI), chronic comorbidities, ethnicity, Charlson Comorbidity Index, Sequential Organ Failure Assessment (SOFA), Acute Physiology Score III (APS III), Simplified Acute Physiology Score II (SAPS II), the minimum Glasgow Coma Scale (GCS) at ICU admission, the mean vital sign and arterial blood gas values obtained between 6 h preceding and 24 h within the ICU admission, as well as outcome measures including intubation rate, 48-h intubation rate, 30-day intubation rate, in-hospital mortality, hospital 28-day mortality, ICU 28-day mortality and the length of stay (LOS) in hospital and ICU.

### Baseline characteristics and clinical outcomes of patients between the HFNC and NIV group

Baseline characteristics and clinical outcomes of the HFNC and NIV therapy groups from MIMIC IV were used as the dataset for the cross-sectional analysis. The detailed comparisons included the variables of the general materials, physiological parameters for the first ICU day, and clinical outcomes (mortality, ntubation rate, and LOS in hospital and ICU).

### Survival analysis of the 48-h and 30-day intubation rate

After cross-sectional analysis, we performed the Kaplan–Meier curves for further survival analysis of the 48-h and 30-day intubation rate between the two groups.

### The 48-h intubation predictive model construction and validation

After randomization, 70% of all eligible patients in the MIMIC IV database were used as the dataset for model construction and 30% as the internal validation set. All eligible patients in the MIMIC III database serve as an external validation of the model. After baseline information comparison for the assessment of distribution consistency, nine machine learning (Support Vector Machine, Neural Network, K nearest neighbor, Decision tree, quadratic discriminant analysis, naive Bayes, Linear discriminant analysis, kernel, logistic regression) and six ensemble algorithms (subspace KNN, Bootstrap Random Forest, AdaBooost Tree, GentleBoost Tree, LogitBoost Tree, RUSBooost Tree) were used to train the training dataset using the features illustrated in the previous studies as well as suggested by professional clinicians. The parameters with a missing rate above 40% were not accepted in the final model due to the bias of predictive accuracy (19). After establishing the prediction model through various machine learning methods, we plotted the receiver operating characteristic curve (ROC) of the constructed models. The model with the highest predictive accuracy, as assessed by the area under the curve (AUROC), threshold, sensitivity, specificity, and Youden index was selected as the best model.

### Model interpretability

Based on the optimal predictive model of AUROC, we calculated the Shapley value of the optimal model and drew the Shapley Explanation plot (12). We used the Shapley additive interpretation algorithm to identify the five characteristic variables of promoting or inhibiting outcomes to determine the risk factors for 48-h intubation.

### Statistical analysis

In the data preprocessing stage, the original data outliers and missing values were filled and interpolated using Matlab dataCleaner APP. The outliers were determined and processed using the Tukey’s test and clinical experts’ advice. Linear interpolation was then used to fill in the identified outliers. The missing values were filled using the nearest neighbor method, and the data of different dimensions were normalized using the extreme value method (left limit is 0 and right limit is 1). To address the class imbalance issue in the test set, the Synthetic Minority Over-Sampling Technique (SMOTE) was applied to improve model generalization.

Following the Kolmogorov–Smirnoff test results, continuous variables were expressed as means and standard deviation when normally distributed and compared using an independent samples t-test or as medians and interquartile range compared using the Mann–Whitney test otherwise. Categorical variables were described as frequencies and percentages and were compared using the Chi-squared test or Fisher’s exact test to compare proportions. The prognostic analysis between the two therapy groups was performed by Kaplan-Meier curves using log-rank test. All tests were two-tailed, and differences were considered statistically significant when *p* < 0.05.

During the model construction stage, nine machine learning and six ensemble algorithms were used to model the training dataset, with 10-fold cross-validation to enhance prediction accuracy. The constructed models were evaluated by AUROC, threshold, sensitivity, specificity, and Youden index in both internal and external validation sets. Finally, the model with the best performance was selected based on the above evaluation criteria. Shapley values were calculated and a Shapley explanation plot was produced to quantify the contribution of the 10 most important features and the explainability of an individual observation in the optimal model.

The data processing, statistical analyses, and predictive construction were performed using R (version 4.2.2) and Matlab software (R2022b Version, MathWorks Corporation, United States).

## Results

### Patient inclusion and characteristics of general materials

A total of 123,042 distinct hospital admissions (*n* = 53423 for MIMIC III, *n* = 69619 for MIMIC IV) were screened of which, 673 from the MIMIC IV database and 408 from the MIMIC III database were finally included. A total of 363 patients who received HFNC and 310 who received NIV as initial therapy were included in the MIMIC-IV for prognostic analysis. There were no significant differences in patient gender and age between the HFNC and NIV groups. The BMI (27.4 vs. 33.8, *p* < 0.001); the SAPS II scores (37 vs. 39, *p* = 0.005); and the proportion of chronic complications such as coronary artery disease (23.7% vs. 42.6%, p < 0.001), chronic obstructive pulmonary disease (COPD, 16.0% vs. 26.5%, *p* = 0.001), and diabetes (30.0% vs. 46.8%, *p* < 0.001) were higher in the NIV group, which indicated a more complex clinical situation compared with the HFNC group. More characteristics of general materials in the test dataset and validation datasets are shown in Tables 1, 2.

**Table 1:** Baseline characteristics of general materials between the HFNC and NIV group.

	MIMIC IV		Z/χ^2^	p
	HFNC (*n* = 363)	NIV (*n* = 310)		
Demographic information				
Male, n(%)	217 (59.8)	190 (61.3)	0.160	0.689
Age, median [IQR], year	68.5 [58.2,80.1]	67.4 [60,1,77.0]	−0.957	0.339
BMI, median [IQR], kg/m^2^	27.4 [23.9,31.9]	33.8 [28.2,40.1]	7.600	<0.001
Ethnicity, n(%)			8.160	0.086
White	247 (68.0)	221 (71.3)		
Black	27 (7.4)	34 (11.0)		
Hispanic/Latino	20 (5.5)	7 (2.3)		
Asian	9 (2.5)	6 (1.9)		
Other	60 (16.5)	42 (13.6)		
Insurance, n(%)			1.260	0.533
Medicaid	25 (6.9)	15 (4.8)		
Medicare	187 (51.5)	164 (52.9)		
Other	151 (41.6)	131 (42.3)		
First ICU, n(%)			35.932	<0.001
CVICU	52 (14.3)	100 (32.3)		
CCU	36 (9.9)	34 (11.0)		
MICU	93 (25.6)	66 (21.3)		
M/SICU	89 (24.5)	52 (16.8)		
SICU	47 (13.0)	22 (7.1)		
TSICU	43 (11.9)	33 (10.7)		
Comorbidity, n(%)				
CAD	86 (23.7)	132 (42.6)	27.242	<0.001
CHF	32 (8.8)	37 (11.9)	1.769	0.184
HBP	80 (22.0)	58 (18.7)	1.137	0.286
COPD	58 (16.0)	82 (26.5)	11.133	0.001
Diabetes	109 (30.0)	145 (46.8)	19.957	<0.001
Stroke	21 (5.8)	8 (2.6)	4.164	0.041
CKD	36 (9.9)	24 (7.7)	0.974	0.324
Scores, median [IQR]				
SOFA	6 [4,8]	6 [4,8]	0.607	0.544
APS III	51 [38,67]	49 [37,64]	−1.440	0.150
SAPS II	37 [29,45]	39 [31,47]	2.833	0.005
Charlson Comorbidity Index	6 [4,8]	6 [4,9]	0.176	0.860
GCS	14 [10,15]	14 [11,14]	−0.208	0.835

IQR, interquartile range. BMI, body mass index; ICU, intensive care unit; CVICU, cardiovascular intensive care unit; CCU, coronary heart disease intensive care unit; MICU, medical intensive care unit; M/SICU, medical/surgical intensive care unit; SICU, surgical intensive care unit; TSICU, trauma surgical intensive care unit; CAD, coronary artery disease; CHF, chronic heart failure; HBP, hypertension blood pressure; COPD, chronic obstructive pulmonary disease; CKD, chronic kidney disease; SOFA, Sequential Organ Failure Assessment; APS III, Acute Physiology Score III; SAPS II, Simplified Acute Physiology Score II; GCS, Glasgow Coma Scale.

**Table 2:** Baseline characteristics of physiological and clinical outcomes between the HFNC and NIV group.

	MIMIC IV		Z/χ^2^	*p*
	HFNC (*n* = 363)	NIV (*n* = 310)		
Panel A Baseline physiological parameters on ICU first day				
HR_mean_, median [IQR], (bmp)	91.2 [81.5,102.9]	85.8 [77.1,97.6]	−3.991	<0.001
RR_mean_, median [IQR], (breath/min)	22.4 [19.3,25.6]	19.6 [17.2,22.5]	−7.067	<0.001
MAP_mean_, median [IQR], (mmHg)	77.5 [70.8,84.9]	76.1 [70.7,82.5]	−1.361	0.174
SpO_2mean_, median [IQR], (%)	95.4 [94.3,96.9]	96.0 [94.3,97.1]	1.753	0.080
PFratio_min_, median [IQR], (mmHg)	142.9 [117.1,180]	170.5 [134,222]	6.067	<0.001
PFratio_max_, median [IQR], (mmHg)	174 [134,241]	243.1 [187.5,306.7]	8.716	<0.001
PaCO_2max_, median [IQR], (mmHg)	45 [37,54]	54 [46,74]	9.424	<0.001
PaCO_2min_, median [IQR], (mmHg)	38 [32,45]	41 [35,54]	5.552	<0.001
PH_min_, median [IQR]	7.37 [7.29,7.43]	7.30 [7.23,7.35]	−9.095	<0.001
PH_max_, median [IQR]	7.42 [7.38,7.47]	7.41 [7.36,7.45]	−4.125	<0.001
Lactate_min_, median [IQR], (mmol/L)	1.3 [1.0,1.8]	1.2 [0.9,1.6]	−2.936	0.003
Lactate_max_, median [IQR], (mmol/L)	1.7 [1.2,2.6]	1.8 [1.2,2.6]	0.162	0.871
SO_2min_, median [IQR], (%)	95 [92,97]	94 [91,97]	−1.867	0.062
Panel B Clinical outcome				
Intubation rate, n(%)	84 (23.1)	50 (16.1)	5.155	0.023
Intubation rate in 48 h, n(%)	58 (16.0)	41 (13.2)	1.009	0.315
Intubation rate in 30 days, n(%)	70 (21.7)	87 (24.9)	0.953	0.329
Mortality at hospital 28 days, n(%)	59 (16.3)	35 (11.3)	3.428	0.064
Mortality at ICU 28 days, n(%)	67 (18.5)	36 (11.6)	6.043	0.014
Mortality in hospital, n(%)	71 (19.6)	37 (11.9)	7.214	0.007
Hospital LOS, median [IQR], (days)	11.0 [6.3,20.4]	8.8 [5.8,15.0]	−2.907	0.004
ICU LOS, median [IQR], (days)	3.7 [2.3,6.0]	2.9 [1.5,5.1]	−4.565	<0.001

IQR, interquartile range. HR_mean_, RR_mean_, MAP_mean_, SpO_2mean_ represent the average heart rate, respiratory rate, mean arterial pressure, peripheral capillary oxygen saturation of the first ICU day; PFratio_min_, PaCO_2min_, PH_min_, Lactate_min_, SO_2min_ represent the minimum value of the PaO_2_/FiO_2_ ratio, PH, lactate, arterial oxygen saturation of the first ICU day; PFratio_max_, PaCO_2max_, PH_max_, Lactate_max_ represent the maximum value of the PaO_2_/FiO_2_ ratio, PaCO_2_, PH, lactate of the first ICU day; LOS represents the length of stay.

### Baseline physiological characteristics and clinical outcomes between the HFNC and NIV groups

As per the physiological parameters illustrated in Table 1A, the mean heart rate (HR_mean_, 91.2 vs. 85.8, *p* < 0.001) and respiratory rate (RR_mean_, 22.4 vs. 19.6, *p* < 0.001) were higher for the first ICU day and the minimum and maximum PaO_2_/FiO_2_ ratio was lower in the HFNC group, which implied that the oxygenation dysfunction was the prominent problem in the HFNC group. The maximum of PaCO_2_ (PaCO_2max_, 45 vs. 54, p < 0.001) and the minimum of PaCO_2_ (PaCO_2min_, 38 vs. 41, *p* < 0.001) were higher and the maximum PH (PH_max_) and the minimum PH value during the first day were lower in the NIV group, which indicated a more serious respiratory failure of both oxygenation impairment and ventilation dysfunction in the NIV group.

For outcome comparison, the NIV group intubation rate (23.1% vs. 16.1%, *p* = 0.023), in-hospital mortality (19.6% vs. 11.9%, *p* = 0.007), and ICU 28-day mortality (18.5% vs. 11.6%, *p* = 0.014) were significantly lower and the ICU LOS (3.7 vs. 2.9, *p* < 0.001) and hospital LOS (11.0 vs. 8.8, *p* = 0.004) were shorter in the NIV group using initial Chi-squared and Mann–Whitney U tests (Table 1B).

### Survival analysis of the 48-h and 30-day intubation rate

**Figure 2:**
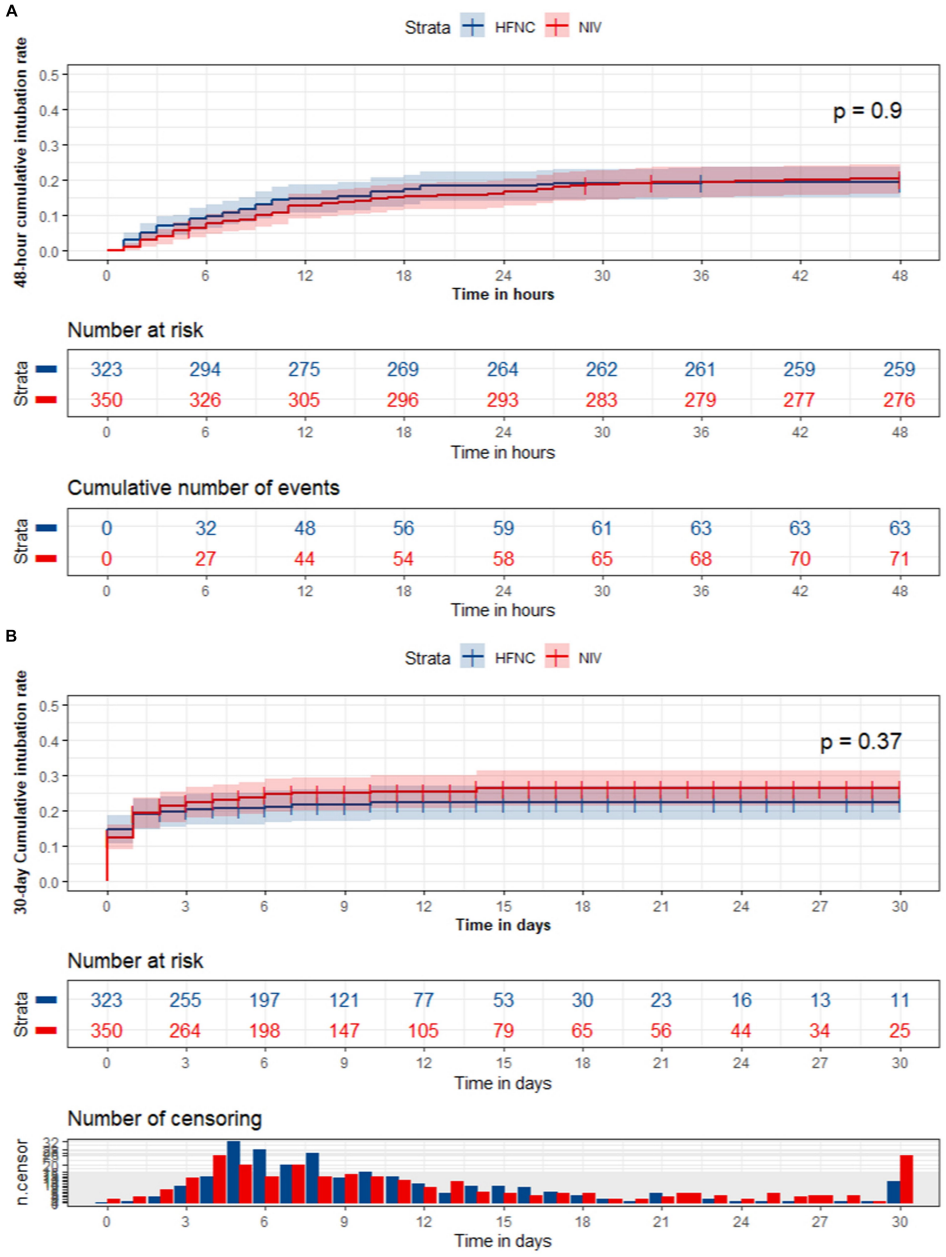
The survival analysis of the 48-hour and 30-day intubation rate between the HFNC and NIV group.

The survival analysis of the 48-h and 30-day intubation rate (all *p* > 0.05) was not significantly different between the HFNC and NIV groups using the Kaplan–Meier curves test (Figure 2).

### The 48-h intubation predictive model construction and validation

Among the tests, the internal and external validation dataset, the proportion of chronic comorbid coronary artery disease (31% vs. 36.1% vs. 26.5%, *p* = 0.045), hypertension (26.1% vs. 26.2% vs. 36.8%, *p* < 0.01), SOFA (6 vs. 6 vs. 5, *p* < 0.001), APS III (50 vs. 55 vs. 46.5, *p* < 0.001), GCS (14 vs. 14 vs. 14, *p* < 0.001), and the ICU LOS (3.7 vs. 3.4 vs. 3.1, *p* = 0.027) were significantly different (Table 3). All the feathers used to train the training dataset were marked as * in (Table 3).

**Table 3:** Baseline characteristics of general materials, physiological, and clinical outcomes between the training, internal, and external validation datasets.

	Datasets			Z/χ^2^	*p*
	Training dataset (*n* = 471)	Internal dataset (*n* = 202)	External dataset (*n* = 408)		
Demographic information					
Male, n(%)*	265(0.6)	125(0.5)	223(0.5)	4.645	0.098
Age, median [IQR], year*	68.4 [59.8,79.3]	69.5 [60.3,78.7]	68.4[59.8,78.1]	0.265	0.876
BMI, median [IQR], kg/m^2^	29.0 [24.5,37.0]	30.1 [24.8,35.7]	29.5[26.0,35.3]	0.543	0.762
Ventilation Status, n (%)				1.723	0.422
HFNC	219 (46.5)	104 (51.5)	203(49.8)		
NIV	252 (53.9)	98 (48.5)	205(50.2)		
Comorbidity, n(%)*					
CAD	146 (31.0)	73 (36.1)	108 (26.5)	6.207	0.045
CHF	51 (10.8)	20 (9.9)	55 (13.5)	2.237	0.327
HBP	123 (26.1)	53 (26.2)	150 (36.8)	13.585	<0.01
COPD	98 (20.8)	35 (17.3)	67 (16.4)	3.016	0.221
Diabetes	188 (39.9)	72 (35.6)	142 (34.8)	2.699	0.259
Stroke	25 (5.3)	7 (3.5)	9 (2.2)	5.838	0.054
CKD	38(8.1)	17 (8.4)	28(6.9)	0.727	0.639
Scores, median [IQR]					
SOFA*	6 [4,8]	6 [4,9]	5 [4,7]	22.995	<0.001
APS III*	50 [39,65.8]	55 [40,70]	46.5 [36,58]	21.089	<0.001
SAPS II*	38 [30,48]	41 [33,49]	39 [31,48]	2.062	0.357
Charlson Comorbidity Index*	6 [4,9]	7 [4,9]	6 [4.5,8]	0.761	0.684
GCS*	14 [10,15]	14 [10,15]	14 [12,15]	14.608	<0.001
Panel A Baseline physiological parameters on ICU first day					
HR_mean_*, median [IQR], (bmp)	88.0 [79.2,99.0]	90.9 [79.3,100.8]	88.8 [79.2,101.4]	1.191	0.551
RR_mean_*, median [IQR], (breath/min)	21.3 [18.1,24.9]	21.1 [18.6,24.2]	21.2 [18.4,24,2]	0.121	0.941
MAP_mean_*, median [IQR], (mmHg)	75.6 [69.9,83.0]	76.1 [70.3,83.3]	77 [70.4,83.6]	1.070	0.586
SpO_2mean_*, median [IQR], (%)	95.8 [94.2,97.2]	95.9 [94.4,97.1]	95.9 [94.5,97.1]	0.849	0.654
PFratio_min_*, median [IQR], (mmHg)	158 [125.7,208]	164.1 [128.0,206.0]	161.8 [122.7,202.9]	0.978	0.613
PaO_2min_*, median [IQR], (mmHg)	68[52,94]	71[46,96]	69[47,91]	2.370	0.306
PaCO_2max_*, median [IQR], (mmHg)	50[42,65]	48 [40,64]	50 [41,68.5]	2.941	0.230
PH_min_*, median [IQR]	7.32 [7.25,7.39]	7.32 [7.26,7.38]	7.33 [7.26,7.40]	1.041	0.594
PH_max_*, median[IQR]	7.41 [7.36,7.46]	7.41 [7.36,7.46]	7.42 [7.36,7.46]	0.086	0.958
Lactate_min_*, median [IQR], (mmol/L)	1.2 [0.9,1.6]	1.2 [1.0,1.5]	1.2[0.9,1.7]	0.329	0.848
Lactate_max_*, median [IQR], (mmol/L)	1.7 [1.2,2.5]	1.6 [1.1,2.6]	1.7[1.1,2.4]	0.380	0.827
SaO_2min_, median [IQR], (%)*	95.5 [92,97]	95.7 [91,97]	95.6[92,97]	1.190	0.552
Panel B Clinical outcome					
Intubation rate, n (%)	116.0 (24.6)	50.0(24.8)	98.0 (24.0)	0.059	0.971
Intubation rate in 48 h, n (%)	93 (19.7)	41 (20.3)	86 (21.1)	0.240	0.887
Mortality at hospital 28 days, n (%)	69 (14.6)	30 (14.9)	51(12.5)	1.043	0.594
Mortality at ICU 28 days, n (%)	75 (15.9)	33 (16.3)	56(13.7)	1.083	0.582
Mortality in hospital, n (%)	79 (16.8)	35 (17.3)	58(14.2)	1.441	0.487
Hospital LOS, median [IQR], (days)	9.9 [6.3,16.5]	10.3 [6.0,17.1]	9.3 [5.7,15.3]	3.301	0.192
ICU LOS, median [IQR], (days)	3.7 [2.2,6.6]	3.4 [1.9,6.0]	3.1 [1.9,5.21]	7.220	0.027

Data are presented as median [interquartile range] or number (%). BMI represents body mass index; ICU represents intensive care unit; CAD represents coronary artery disease; CHF represents chronic heart failure; HBP represents hypertension blood pressure; COPD represents chronic obstructive pulmonary disease; CKD represents chronic kidney disease; SOFA represents Sequential Organ Failure Assessment; APSIII represents Acute Physiology Score III; SAPS II represents Simplified Acute Physiology Score II; HR_mean_, RR_mean_, MAP_mean_, SpO2_mean_ represent the average heart rate, respiratory rate, mean arterial pressure, peripheral capillary oxygen saturation of the first ICU day; PFratio_min_, PH_min_, Lactate_min_, SO2_min_ represent the minimum value of the PaO_2_/FiO_2_ ratio, PH, lactate, arterial oxygen saturation of the first ICU day; PaCO_2max_, PH_max_, Lactate_max_ represent the maximum value of the PaCO_2_, PH, lactate of the first ICU day; LOS represents the length of stay. * marked as parameters for model construction of 48-h intubation.

**Figure 3:**
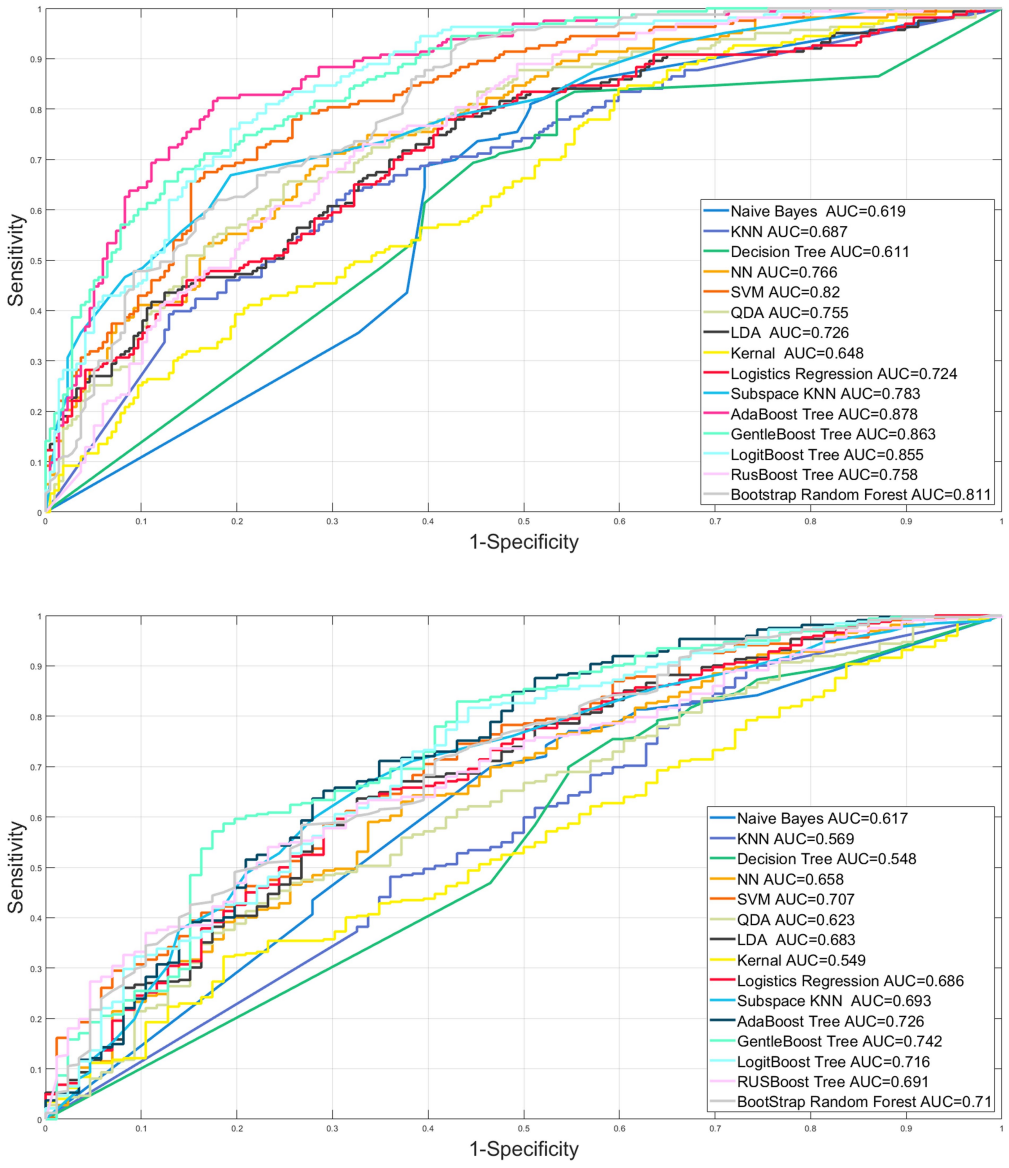
Internal and external validation of the machine learning models.

The AUROC of nine machine learning (Support vector machine, Neural network, K nearest neighbor, Decision tree, quadratic discriminant analysis, Naive Bayes, Linear discriminant analysis, kernel, logistic regression) and six ensemble algorithms (subspace KNN, Bootstrap Random Forest, AdaBooost Tree, GentleBoost Tree, LogitBoost Tree, RUSBooost Tree) in the internal validation set were 0.820, 0.766, 0.687, 0.611, 0.755, 0.619, 0.726, 0.648, 0.724, 0.783, 0.811, 0.878, 0.863, 0.855, and 0.758, respectively. The AUROC in the external validation set was 0.707, 0.658, 0.569, 0.548, 0.623, 0.617, 0.683, 0.549, 0.686, 0.693, 0.710, 0.726, 0.742, 0.716, and 0.691, respectively (Table 4; Figure 3).

**Table 4:** Model performance in the internal and external validation datasets.

Models	Internal validation					External validation				
	AUC	thresholds	Sensitivity	Specificity	Youden index	AUC	thresholds	Sensitivity	Specificity	Youden index
Naive Bayes	0.619 [0.565,0.673]	0.200	0.525	0.748	0.274	0.617 [0.553,0.681]	0.947	0.442	0.770	0.212
KNN	0.687 [0.631,0.739]	0.611	0.622	0.675	0.297	0.569 [0.496,0.636]	0.318	0.430	0.677	0.107
Decision Tree	0.611 [0.557,0.669]	0.074	0.493	0.724	0.217	0.548 [0.496,0.615]	0.800	0.360	0.786	0.239
NN	0.766 [0.717,0.810]	0.882	0.576	0.779	0.355	0.658 [0.586,0.717]	0.987	0.360	0.826	0.187
SVM	0.820 [0.768,0.858]	0.053	0.682	0.804	0.486	0.707 [0.645,0.765]	0.092	0.465	0.795	0.260
QD	0.755 [0.703,0.800]	0.853	0.548	0.810	0.358	0.622 [0.556,0.683]	0.947	0.337	0.795	0.132
LD	0.726 [0.671,0.775]	0.365	0.687	0.607	0.294	0.683 [0.619,0.747]	0.549	0.605	0.680	0.285
Kernal	0.648 [0.597,0.703]	−0.213	0.465	0.699	0.165	0.549 [0.482,0.617]	0.763	0.372	0.665	0.037
logistic	0.724 [0.667,0.773]	0.360	0.677	0.607	0.285	0.686 [0.621,0.749]	0.555	0.558	0.683	0.241
Subspace KNN	0.783 [0.731,0.824]	0.567	0.645	0.736	0.381	0.693 [0.628,0.755]	0.567	0.512	0.761	0.107
AdaBoost Tree	0.878 [0.837,0.909]	7.916	0.687	0.883	0.570	0.726 [0.665,0.789]	7.553	0.360	0.919	0.280
GentleBoost Tree	0.863 [0.823,0.896]	0.029	0.650	0.865	0.515	0.742 [0.682,0.798]	0.054	0.384	0.904	0.287
LogitBoost Tree	0.855 [0.813,0.889]	4.433	0.668	0.883	0.552	0.716 [0.654,0.777]	5.810	0.326	0.913	0.212
RUSBoost Tree	0.758 [0.708,0.807]	2.342	0.530	0.847	0.377	0.691 [0.632,0.747]	2.880	0.279	0.888	0.167
Bootstrap Random Forest	0.811 [0.763,0.849]	0.412	0.622	0.816	0.438	0.710 [0.649,0.770]	0.715	0.349	0.882	0.231

AUC, area under curve; Youden index was defined as sensitivity + specificity-1. KNN, K-Nearest Neighbor; NN, neural network; SVM, support vector machine; QDA, quadratic discriminant analysis; LDA, linear discriminant analysis; Subspace KNN, subspace K-Nearest Neighbor; AdaBoost, adaptive boosting; RUSBoost, random under-sampling boosting.

**Figure 4:**
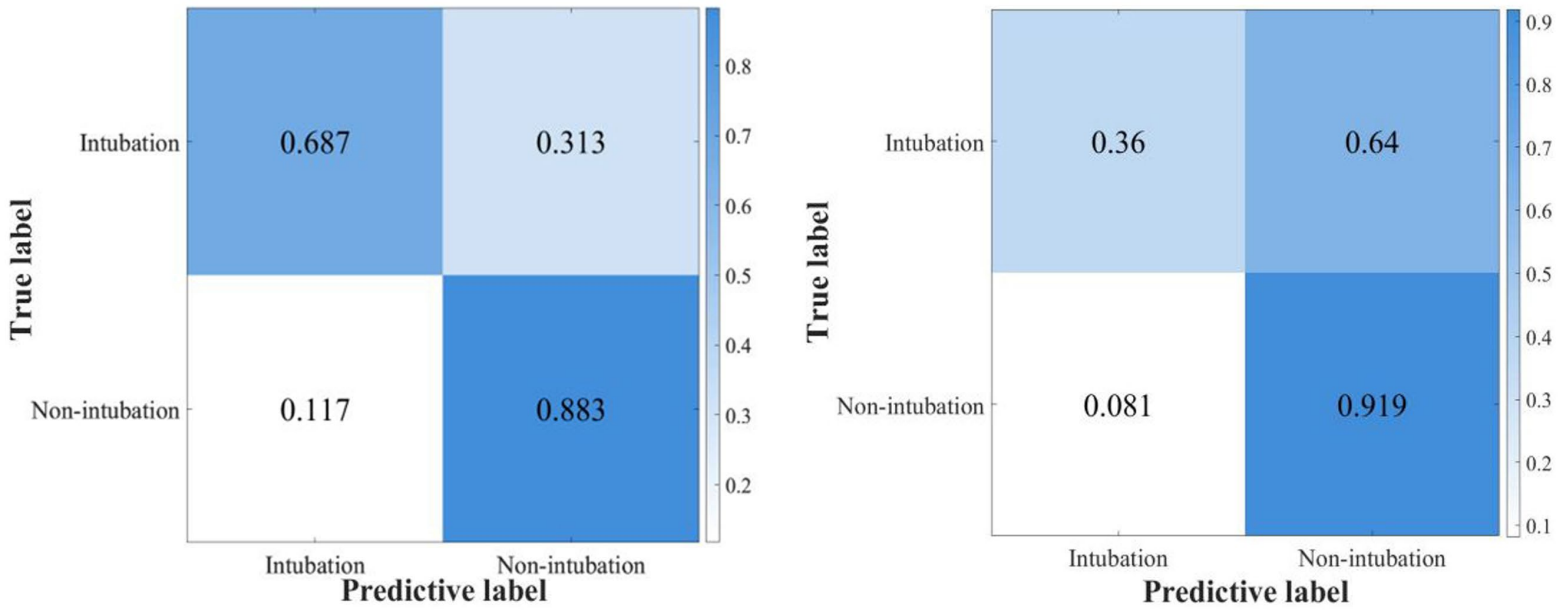
The confusion matrix of the ensemble AdaBoost decision tree model for internal and external validation.

The confusion matrix of the best AdaBoost decision tree model based on the best AUROC (the optimal hyperparameter is maximum split 54, number of learners 413, learning rate 0.9194) is shown in Figure 4.

### Shapley value

**Figure 5:**
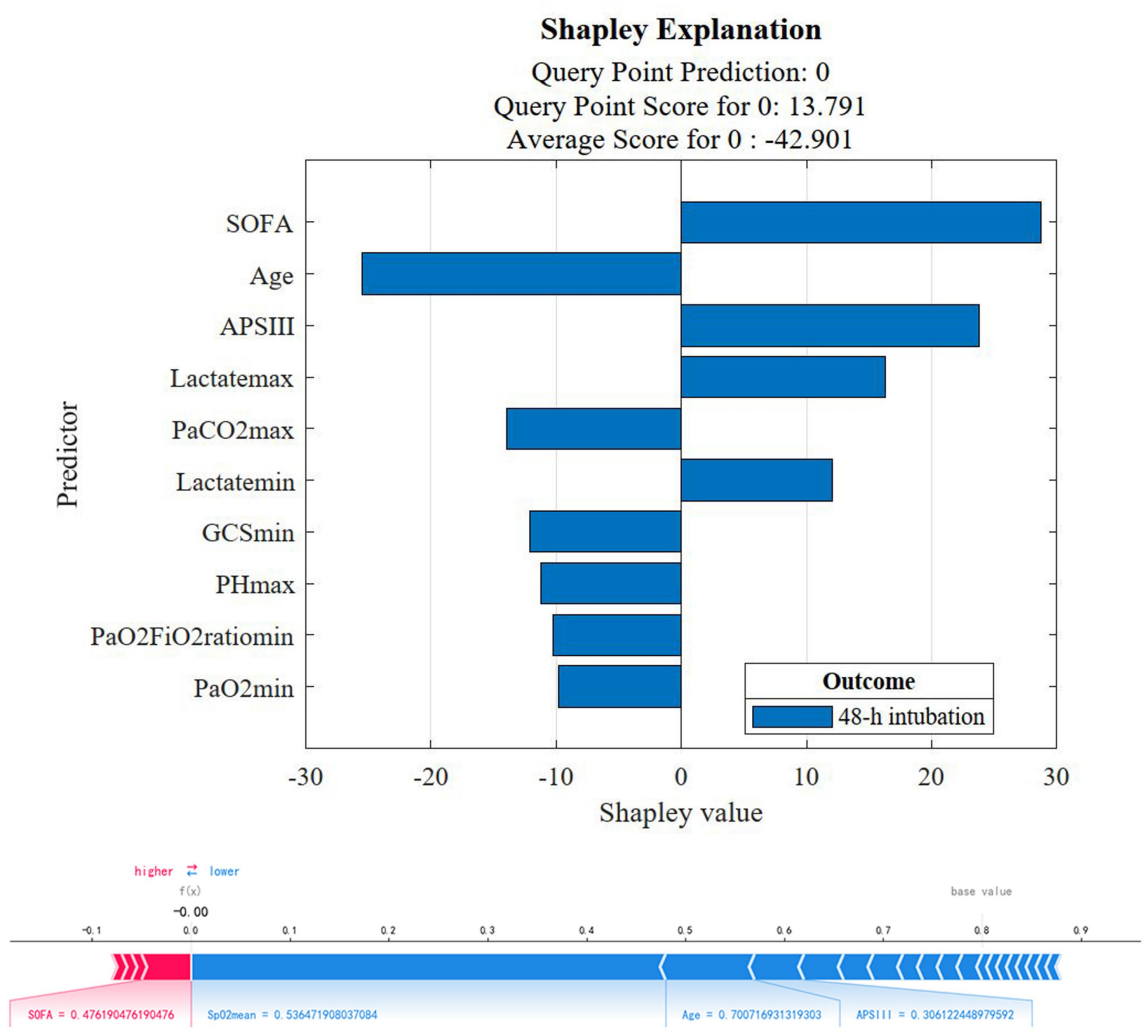
The Shapley Explanation plot for the ensemble AdaBoost Tree model.

Based on the best model to show the SOFA score, APS II score, the maximum and minimum values of lactate as the risk factors of 48-h intubation, age, maximum PaCO_2_ value (PaCO2_max_), GCS, PH_max_, and the minimum value of PaO_2_/FiO_2_ ratio (PaO_2_/FiO_2min_) and PaO_2_ (PaO_2min_) are protective factors for 48-h intubation (Figure 5 upper graph). The individual predictive plot showed the explainability of the optimal model for individual observation (Figure 5 lower graph).

## Discussion

In our study, more than 120,000 patients from 2001 to 2019 were screened from MIMIC III and IV databases. A total of 673 eligible patients from MIMIC IV were included in the prognostic analysis and 48-h intubation model construction and internal validation, while 408 eligible patients from MIMIC III were included in the external validation. We found that (1) the NIV group intubation rate, ICU 28-day mortality, and in-hospital mortality were significantly lower and the lengths of stay in the ICU and hospital were shorter compared with the HFNC group in cross-sectional analysis; (2) after considering time effect, the initial therapy of either HFNC or NIV had no significant influence on the total and 48-h intubation rate; (3) the ensemble AdaBoost Tree algorithm (internal and external validation set AUROC 0.878, 0.726) was the best model in the validation cohort, providing a proper method for clinicians to make clinical decisions and a reference for researchers to optimize the models in further prospective studies; (4) The model Shapley algorithm showed SOFA, APSIII, the minimum and maximum lactate value as risk factors for early failure and age, the PaCO_2max_ and PH_max_, GCS, PaO_2_/FiO_2min,_ and PaO_2min_ value as protective factors.

HFNC, with a high concentration of oxygen continuously flushing physiological dead space (20, 21), low level of positive end-expiratory pressure (22), and increased patient comfort (23) was a frequently used non-invasive equipment for improving oxygenation. NIV was another commonly used non-invasive respiratory support for enhancing gas exchange and ventilation. It mainly improves oxygenation through three mechanisms: moderate inspiratory pressure to enhance ventilation (24, 25), adjustable end-expiratory positive pressure (25), and decreased left ventricular afterload to enhance left ventricular function (24). In comparison to HFNC, NIV can provide higher airway pressure for ventilation support, especially with helmet NIV (11). These differences explained why professional clinicians were inclined to select HFNC as a therapy for single hypoxemia and select NIV as a therapy for complex hypoxemia combined with coronary artery disease, COPD, or respiratory acidosis in our baseline information comparison of two groups in the baseline clinical parameters comparison.

The NIV group was superior to the HFNC group in terms of the total intubation rate, ICU 28-day mortality, in-hospital mortality, ICU LOS, and hospital LOS in the cross-sectional analysis. Our primary outcome was that the 48-h and 30-day intubation rates were not significantly different between the groups after considering the factor of time to the endpoint event in the survival analysis, which reinforced that the difference may be due to the heterogeneity of baseline information of the groups. These results are consistent with the consensus on acute hypoxemic failure treated by HFNC or NIV (10, 26, 27). In 2020, Ferreyro (28) et al. conducted a network meta-analysis on endotracheal intubation of non-invasive oxygenation strategies with acute hypoxemic respiratory failure. They found that helmet NIV was associated with a decreased risk of endotracheal intubation compared with HFNC (RR, 0.35; absolute risk difference, −0.20; low certainty) and face mask non-invasive ventilation (RR, 0.35; absolute risk difference, −0.20; low certainty), and there was no significant difference between face mask NIV and HFNC (RR, 1.01; absolute risk difference, −0.00; low certainty). In 2022, Perkins et al. (29) performed a multicenter random multicenter random control trial comparing continuous positive airway pressure (CPAP) and HFNC with conventional oxygen therapy (COT) in COVID-19 patients continuous positive airway pressure (CPAP), HFNC, and conventional oxygen therapy (COT) in COVID-19 patients with acute hypoxemic respiratory failure. They found that the intubation rate within 30 days was significantly lower with CPAP vs. COT (36.3% vs. 44.4%, absolute difference, −8%, *p* = 0.03) but was not significantly different between HFNC and COT (44.3% vs. 45.1%, absolute difference, −1%, *p* = 0.83). Therefore, the different physiopathological mechanisms of primary disease and therapy parameters may be important factors in influencing treatment failure rate. More disease states and detailed treatment parameters need to be controlled in future studies.

Machine learning, as an essential part of artificial intelligence, can analyze complex and diverse medical data using various algorithms in data mining and analysis. It can provide early warning and support for medical clinical decision-making. In the electronic health information system of intensive care units, machine learning and deep learning can perform better than traditional models or single indicators in processing nonlinear, dynamic medical data with complex correlation, especially with high granularity monitoring systems collecting continuous data on respiratory, hemodynamic, neurological, and clinical variables. In previous studies, the traditional risk assessment model for non-invasive supportive therapy failure and independent risk factors were HACOR score to dynamically assess the risk of intubation in mask NIV patients (30) andSpO_2_ / FiO_2_ to assess respiratory rate ratio (ROX) (31) and ROX / HR (32) in HFNC patients; esophageal pressure fluctuation (14); and exhaled tidal volume (15, 16) in NIV patients, etc. Due to the inherent deficiency of using algorithms, these traditional models and indicators mainly focus on the physiological parameters before or after treatment and do not include the impact of primary disease, the severity of the organ dysfunction before treatment, and the treatment-related parameters. Therefore, machine learning methods that combine multiple types of complex parameters when handling similar tasks may be more competent. In 2020, Siu et al. (33) conducted a retrospective analysis of the MIMIC III and eICU databases to construct a 24-h ICU admission intubation predictive model, using logistics regression (AUC 0.77) and random forest algorithm (AUC 0.86). In 2021, Arvind et al. (34) conducted a retrospective analysis based on medical data from 4,087 adult patients who were hospitalized with confirmed COVID-19 or under suspected medical observation in five New York hospitals. The team compared the predictive accuracy of the random forest model and the ROX index in 72-h endotracheal intubation, respectively. Random forests had a better predicted performance (mean AUC 0.84) than the ROX index (mean AUC 0.64). In a retrospective analysis of Shashikumar et al. (35) based on ICU patients at the San Diego Hospital of California University (trial set *n* = 18,528) and Massachusetts General Hospital (validation set *n* = 3,888), a deep learning prediction model of invasive mechanical ventilation (trial set and validation set AUC, 0.895 vs. 0.882) was better than the ROX index (0.738 vs. 0.782). Based on the above results, the predictive efficiency of mechanical learning is generally higher than that of traditional prediction models or single predictive indicators.

In this study, we constructed nine machine learning models and six integrated learning models in the test dataset and compared the prediction efficiency in the internal and external validation sets. The prediction accuracy of all models in the internal validation set is higher than that of the external validation set, which may be partly due to the potential differences among the datasets caused by the different admission time of the original database. Combining the AUROC, sensitivity, specificity, and Youden index of each model, the ensemble AdaBoost decision tree model performed the best. The AdaBoost model, short for Adaptive Boosting, first introduced by Freund and Schapire (36), is a widely used and researched model based on the boosting algorithm. Schapire’s experiments involving 300 rounds of boosting tests showed that AdaBoost often avoids overfitting with excellent and stable prediction performance. The AdaBoost model in the internal validation set has high prediction efficiency (AUC 0.878, sensitivity 0.687, specificity 0.883), and the external validation set has high specificity and relatively low sensitivity (AUC 0.726, sensitivity 0.360, and specificity 0.919). Possible reasons for the substantial difference in the specificity and sensitivity of the external validation set may include the following. 1. The uneven distribution of the modeling data on the outcome factors of intubation makes it easier to identify patients with successful ventilation using the constructed model. Therefore, in constructing the model, we adopted the artificial oversampling method to deal with the category imbalance problem in order to improve the identification ability of a few classes (intubation patients) and increase the generalization ability of the model. 2. The original database did not fully record the parameters related to non-invasive respiratory supportive therapy and therapeutic efficiency assessment. 3. The two datasets are derived from medical databases at different periods, and advances in supportive treatment make it easier for the model constructed in MIMIC IV (2008–2019) to identify successfully ventilated patients in the external validation of the previous MIMIC III (2001–2012) database. However, the high specificity of this model can assist clinicians in accurately screening patients with successful ventilation and can warn medical staff to implement early intervention and preparation of high-failure-risk patients during ventilation and avoid complications related to high-risk emergency intubation and delayed intubation, thus improving patient prognosis. This has been of great clinical significance during the COVID-19 pandemic, with existing medical resources being tight and scarce.

After building the optimal prediction model, we also introduce the game-theoretic Shapley-value method to weigh the importance of each feature and, thus, explain the model predictions. SOFA (37–39), APACHE II (39, 40), and lactate (41, 42) were also confirmed as independent predictors or related factors of tracheal intubation in previous studies. At the same time, elderly and severe patients being more inclined to choose “non-intubation” may be an important reason why age becomes a protective factor (43). In 2015, Correa et al. (42) found a lower PaCO_2_ level in NIV failure patients with acute hypoxic respiratory failure. In 2020, Park et al. (44) illustrated that lower PaCO_2_ levels were an independent predictor of NIV treatment failure, which is consistent with the analysis in our study of PaCO_2_ as a protective factor for treatment failure. In 2012, Nicolini et al. (45) illustrated that the baseline oxygenation indicator PaO_2_/FiO_2_ ratio ≤ 127 was an independent predictor of tracheal intubation in patients with acute hypoxemic respiratory failure caused by H1N1. In 2018, Frat et al. (16) used a multicenter study of acute noninvasive respiratory support patients with hypoxic respiratory failure to confirm PaO_2_/FiO_2_ ≤ 200 as an independent risk factor for tracheal intubation. In 2021, Teresa et al. (46) found in COVID-19 patients with NIV failure, the PaO_2_, PaO_2_ /FiO_2_ ratio, and PaCO_2_ value were relatively lower. In addition, the GCS score is also a common clinical scoring standard to determine the state of consciousness of patients, which has also been confirmed to be negatively associated with the risk of endotracheal intubation (16). In a multicenter study by Ricard et al. (38) in 2021, PH was found to be a protective factor (OR 0.47, 95%CI: 0.24–086, *p* = 0.03) for intubation in patients with acute respiratory failure due to COVID-19. Therefore, early organ function support to improve patients’ SOFA and APSII scores, heart rate, PaO_2,_ and lactate values can be useful to reduce the early failure rate and improve patient prognosis.

### Limitations

Several limitations of this study should be considered. Firstly, our study was a retrospective research study, which mainly used the online MIMIC database. Based on the dataset and technical reasons, we did not involve therapy parameters such as treatment duration, interfaces, and treatment settings, which were also important factors that could influence the outcome according to our daily clinical observation. Secondly, due to the large amount of missing data, we also did not include the change values of respiratory treatment parameters and physiological indicators before and after treatment, which may influence the treatment outcome in clinical practice. Finally, important features based on Shapley interpretability analysis must also be validated in randomized controlled trials with large samples. We will further study and explore the following two directions: designing a prospective study cohort to obtain more real-time parameters and further constructing more effective features to optimize the prediction model; designing prospective clinical randomized controlled trials to verify the impact of important feature factors in the risk prediction model in order to improve patient outcomes.

## Conclusion

In conclusion, the NIV group was found to be associated with reduced intubation rate, ICU 28-day and in-hospital mortality, and shorter ICU and length of stay compared with HFNC using cross-sectional analysis. It was also illustrated that the initial ventilation options, either HFNC or NIV therapy, had no significant influence on the 48-h intubation rate after considering the time effect and other confounding factors. The ensemble AdaBoost decision tree model may assist clinicians in making clinical decisions, and early organ function support to improve patients’ SOFA and APSII scores, heart rate, PaO_2,_ and lactate values can be used to reduce the early failure rate and improve patient prognosis.

## Data availability statement

The datasets presented in this study can be found in online repositories. The names of the repository/repositories and accession number(s) can be found at: MIMIC-IV repository, https://physionet.org/content/mimiciv/1.0/MIMIC-III repository, https://physionet.org/content/mimiciii/1.4/.

## Author contributions

QL and RC conceptualized the research aims and planned the analyses. WF extracted the data from the MIMIC-IV database. XL did the data processing and guided the literature review. LG participated in data analysis and interpretation. ZL, ZH, JN, and QH did the literature search. WF wrote the first draft of the paper. All authors contributed to the article and approved the submitted version.

## Glossary

HFNC: High-flow nasal cannula
NIV: Non-invasive ventilation
ICU: Intensive care unit
MIMIC III/IV: Medical Information Mart for Intensive Care III/IV
BMI: Body mass index
COPD: chronic obstructive pulmonary disease
SOFA: Sequential Organ Failure Assessment
APS III: Acute Physiology Score III
SAPS II: Simplified Acute Physiology Score II
LOS: length of stay
AUROC: area under the receiver operating characteristic curve
PaO_2max_: maximum PaO_2_ on the first ICU day
PaCO_2max_: maximum PaCO_2_ on the first ICU day
PH_max_: maximum PH value on the first ICU day
lactate_min_: minimum lactate value on the first ICU day
lactate_max_: maximum lactate value on the first ICU day
SpO_2mean_: average value of SpO_2_ on the first ICU day
HR_mean_: average value of heart rate on the first ICU day
MAP_mean_: average value of the mean arterial pressure on the first ICU day
KNN: K-Nearest Neighbor
NN: neural network
SVM: support vector machine
QDA: quadratic discriminant analysis
LDA: linear discriminant analysis
Subspace KNN: subspace K-Nearest Neighbor
AdaBoost: adaptive boosting
RUSBoost: random under-sampling boosting
